# Publisher Correction: Double mimicry evades tRNA synthetase editing by toxic vegetable-sourced non-proteinogenic amino acid

**DOI:** 10.1038/s41467-018-03594-1

**Published:** 2018-03-13

**Authors:** Youngzee Song, Huihao Zhou, My-Nuong Vo, Yi Shi, Mir Hussain Nawaz, Oscar Vargas-Rodriguez, Jolene K. Diedrich, John R. Yates, Shuji Kishi, Karin Musier-Forsyth, Paul Schimmel

**Affiliations:** 1 The Scripps Laboratories for tRNA Synthetase Research and the Department of Molecular Medicine, The Skaggs Institute for Chemical Biology, The Scripps Research Institute, 92037 La Jolla, CA USA; 2 Department of Chemistry and Biochemistry, Center for RNA Biology, The Ohio State University, Columbus, OH 43220 USA; 3 Department of Molecular Medicine, The Scripps Research Institute, La Jolla, CA 92037 USA; 4 The Scripps Laboratories for tRNA Synthetase Research and Department of Molecular Medicine, The Scripps Research Institute, Jupiter, FL 33458 USA; 5 Present Address: Research Center for Drug Discovery, School of Pharmaceutical Sciences, Sun Yat-sen University, 510006 Guangzhou, China; 6 Present Address: Department of Chemistry, New York University, PO Box 129188 Abu Dhabi, United Arab Emirates

Correction to: *Nature Communications* 10.1038/s41467-017-02201-z, published online 22 December 2017

In the original version of this Article, extraneous text not belonging to the article was accidentally appended to end of the first paragraph of the discussion. This error has now been corrected in both the PDF and HTML versions of the Article.

