# Supplementary material for: Double mimicry evades tRNA synthetase editing by toxic vegetable-sourced non-proteinogenic amino acid
Source: Nat Commun. 2017 Dec 22;8:2281. doi: 10.1038/s41467-017-02201-z (PMC5741666; doi:10.1038/s41467-017-02201-z)
Supplement: Supplementary file 1 — Supplementary Information [file 41467_2017_2201_MOESM1_ESM.pdf]

Supplementary Information

Supplementary Figures

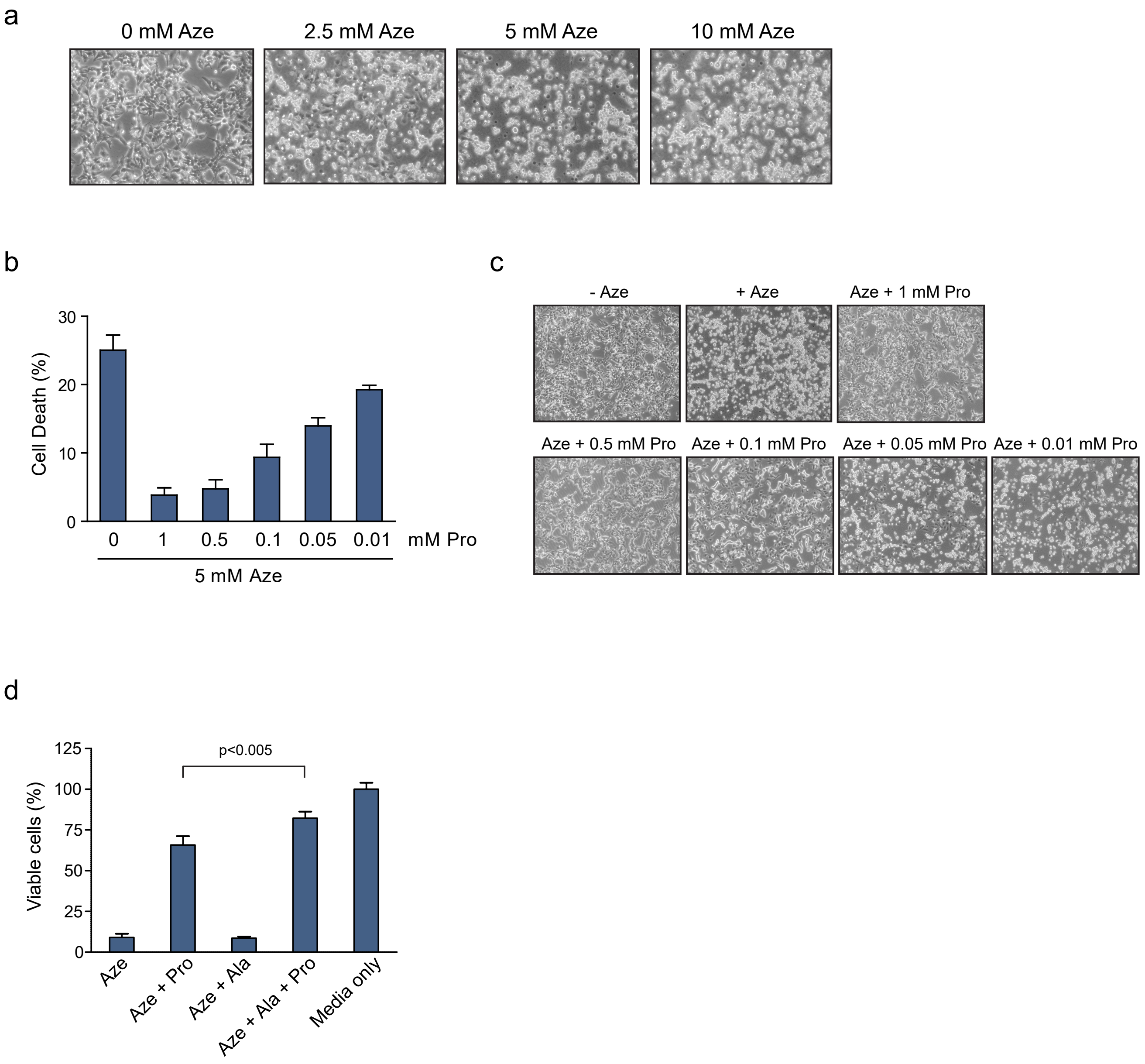

Supplementary Figure 1. Pro rescues Aze toxicity

a. Images of HeLa cells treated with 0-10 mM Aze for 24 hrs. b. HeLa cells were treated with 5 mM Aze and different concentrations (0-1000 μM) of Pro for 24 hrs. Cell death was quantified and plotted. c. Cell images of b. d. At high concentrations of Aze (40 mM), Ala added to Pro can partially rescue HeLa cell toxicity (Pro and Ala concentrations were 1 mM). All error bars represent s.d..

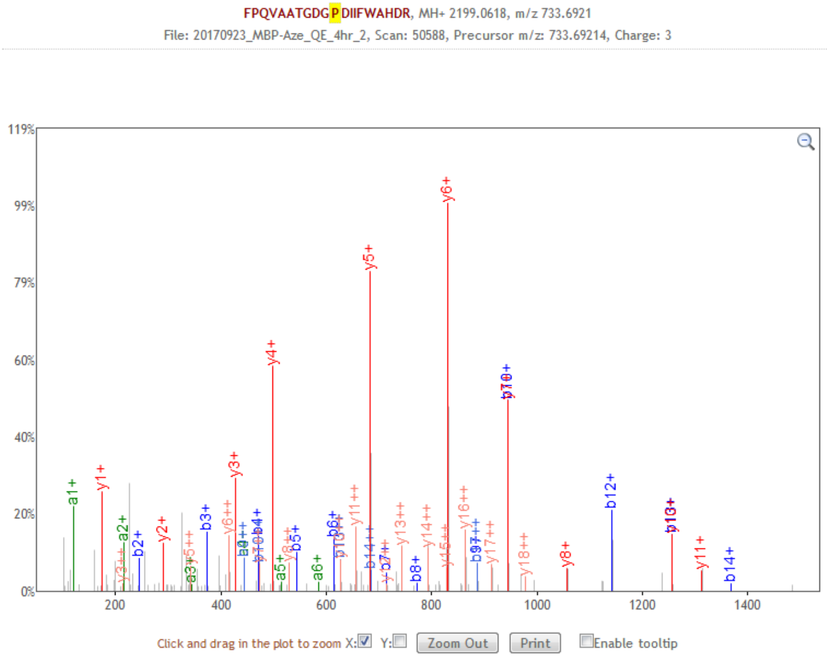

a+	b+	b2+	#	Seq	#	y+	y2+
120.0808	148.0757	74.5415	1	F	20		
217.1335	245.1285	123.0679	2	P	19	2051.9934	1026.5003
345.1921	373.1870	187.0972	3	Q	18	1954.9406	977.9739
444.2605	472.2554	236.6314	4	V	17	1826.8820	913.9446
515.2976	543.2926	272.1499	5	A	16	1727.8136	864.4104
586.3348	614.3297	307.6685	6	A	15	1656.7765	828.8919
687.3824	715.3774	358.1923	7	T	14	1585.7394	793.3733
744.4039	772.3988	386.7030	8	G	13	1484.6917	742.8495
859.4308	887.4258	444.2165	9	D	12	1427.6702	714.3388
916.4523	944.4472	472.7272	10	G	11	1312.6433	656.8253
999.4894	1027.4843	514.2458	11	P	10	1255.6218	628.3146
1114.5164	1142.5113	571.7593	12	D	9	1172.5847	586.7960
1227.6004	1255.5953	628.3013	13	I	8	1057.5578	529.2825
1340.6845	1368.6794	684.8433	14	I	7	944.4737	472.7405
1487.7529	1515.7478	758.3775	15	F	6	831.3896	416.1985
1673.8322	1701.8271	851.4172	16	W	5	684.3212	342.6643
1744.8693	1772.8642	886.9358	17	A	4	498.2419	249.6246
1881.9282	1909.9232	955.4652	18	H	3	427.2048	214.1060
1996.9552	2024.9501	1012.9787	19	D	2	290.1459	145.5766
			20	R	1	175.1190	88.0631

[click] to move table

Static Modifications:
C: 57.02146

Variable Modifications:
P: -14.01565

Supplementary Figure 2. Example of MS/MS spectra showing Aze incorporation at Pro 58 position

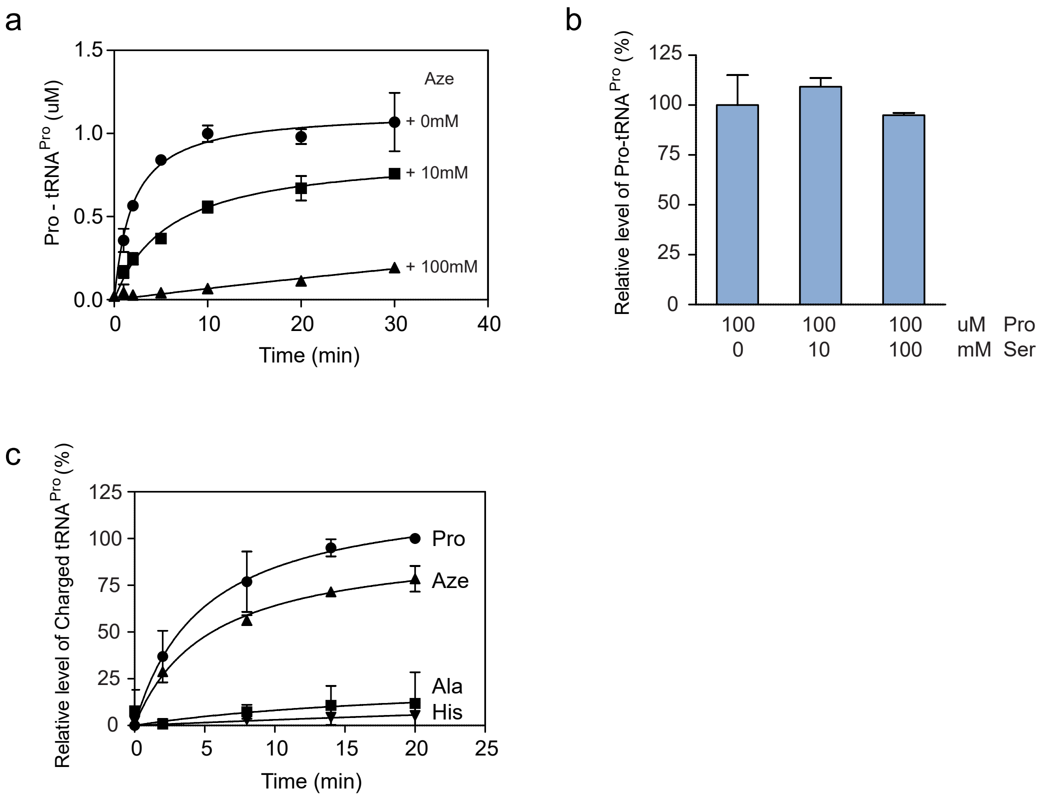

Supplementary Figure 3. *Hs* ProRS charging onto tRNA^{Pro}

a. Time course of Aze competition with Pro charging onto tRNA^{Pro} by *Hs* ProRS. b. Pro-tRNA^{Pro} formation by *Hs* ProRS in the presence of different concentrations of Ser at 20 min (negative control). c. Time course of *Hs* ProRS charging tRNA^{Pro}.

Supplementary Tables

	ProRS-AzeSA	AlaRS-AzeSA
Data collection		
Resolution (Å)	50.0-2.59 (2.63-2.59)[a]	50.0-2.03 (2.14-2.03)
Wavelength (Å)	1.5418	1.5418
Space group	$p3_221$	$c2$
Unit cell parameters a, b, c(Å)	a= b=83.76, c=108.77	a=108.33, b=67.27, c=86.35
Unit cell parameters α, β, γ(°)	α=β=90, γ=120	α=γ=90, β=136.23
Unique reflections	14793 (652)	27565 (3800)
Redundancy	10.2 (8.1)	3.9 (3.7)
Completeness (%)	96.9 (86.5)	99.2 (94.8)
Average I/σ(I)	37.2 (4.8)	15.7 (4.0)
R_{merge}[b] (%)	5.7 (33.7)	6.0 (32.0)
Refinement		
Resolution (Å)	35.54-2.59 (2.66-2.59)	59.7-2.03 (2.09-2.03)
Reflections for refinement/test	13977/749 (931/53)	26163/1390 (1803/93)
R_{work}[c]/R_{free}[d] (%)	22.7/27.2 (29.1/33.5)	18.0/21.5 (25.1/27.5)
RMSD bond (Å)	0.004	0.007
RMSD angle (°)	1.05	1.23
Mean B factor ($Å^2$)	59.4	27.0
Non-hydrogen protein atoms	3749	3046
Water oxygen atoms	26	245
AzeSA atoms	29	29
Zn^{2+}	1	0
MolProbity Ramachandran plot(%)		
Favored regions	98.3	98.2
Allowed regions	100.0	100.0

[a]Values in parentheses are for the highest resolution shell.
[b]$R_{merge} = \Sigma_h \Sigma_l \mid I(h)_l - \langle I(h)\rangle \mid / \Sigma_h \Sigma_l I(h)_l$, where $I(h)_l$ is the lth observation of the reflection h and $\langle I(h)\rangle$ is the weighted average intensity for all observations l of reflection h.
[c]$R_{work}=\sum_h \big| |F_{obs}(h)| - |F_{cal}(h)| \big| / \sum_h |F_{obs}(h)|$, where $F_{obs}(h)$ and $F_{cal}(h)$ are the observed and calculated structure factors for reflection h respectively.
[d]R_{free} was calculated as R_{work} using the 5% of reflections which were selected randomly and omitted from refinement.

Supplementary Table 1. Statistics of X-ray diffraction data collection and structure refinement

	Amino acid	k_{cat} (s^{-1})	K_m (mM)	$k_{cat}\ K_m^{-1}$ ($s^{-1}\ mM^{-1}$)	$k_{cat}\ K_m^{-1}$ (Relative)
Hs ProRS	Pro	33.0 ± 1.2	0.09 ± 0.01	388.6	1
	Aze	15.8 ± 0.7	2.9 ± 0.4	5.5	1 / 71
Hs AlaRS	Ala	9.6 ± 0.3	0.07 ± 0.01	131.4	1
	Aze	4.1 ± 0.2	38.3 ± 5.5	0.11	1 / 1229
	Ser	9.8 ± 0.4	70.8 ± 7.6	0.14	1 / 950

Supplementary Table 2. Kinetic parameters for activation of Pro and Aze by *Hs* ProRS, and Ala and Aze by *Hs* AlaRS

The ATP-PPi exchange assay was carried out at 37 ºC with either 0.05 µM *Hs* ProRS or 0.1 µM *Hs* AlaRS (pH 8.0). For the assays with ProRS, Pro concentrations were 0.03 - 2 mM and Aze concentrations were 1 - 30 mM. For the assays with AlaRS, Ala concentrations were 0.03 - 2 mM, Aze concentrations were 2 - 300 mM and Ser concentrations were 10 - 300 mM.

Non-proteinogenic amino acids	Mimicking amino acids			
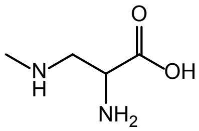<br>BMAA	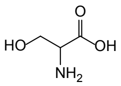<br>Serine	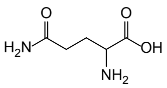<br>Glutamine	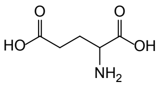<br>Glutamic acid	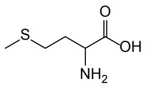<br>Methionine
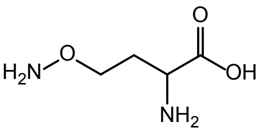<br>Canaline	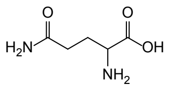<br>Glutamine	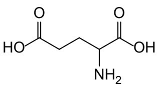<br>Glutamic acid	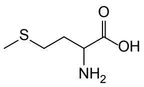<br>Methionine	
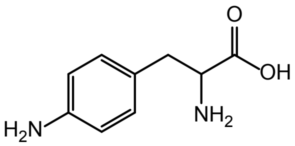<br>p-aminophenylalanine	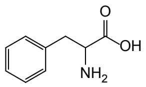<br>Phenylalanine	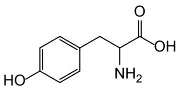<br>Tyrosine		
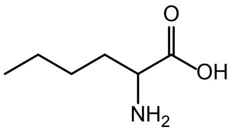<br>Norleucine	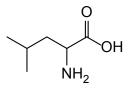<br>Leucine	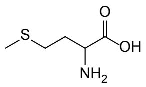<br>Methionine	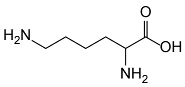<br>Lysine	
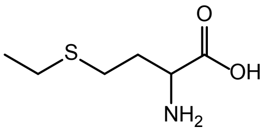<br>Ethionine	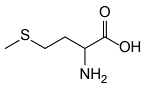<br>Methionine	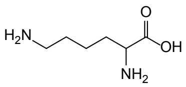<br>Lysine		
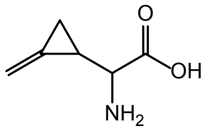<br>A-(methylenecyclopropyl)glycine	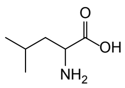<br>Leucine	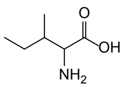<br>Isoleucine		
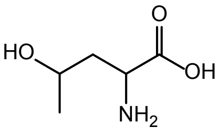<br>G-hydroxynorvaline	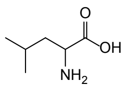<br>Leucine	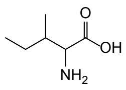<br>Isoleucine		

Supplementary Table 3. Non-proteinogenic amino acids that mimic multiple amino acids
